# Occupational exposures and worker health in decarbonization of energy-intensive industries: a scoping review protocol

**DOI:** 10.3389/fpubh.2026.1933778

**Published:** 2026-08-31

**Authors:** Johanna Samulin Erdem, Ingrid Løken Jørgensen, Lina Wik

**Affiliations:** 1 Department of Occupational Toxicology, National Institute of Occupational Health, Oslo, Norway; 2 National Surveillance for Work Environment and Occupational Health, National Institute of Occupational Health, Oslo, Norway

**Keywords:** decarbonization technologies, energy-intensive industries, green transition, industrial decarbonization, occupational exposure, occupational health, worker health, workplace hazards

## Abstract

Energy-intensive industries account for a substantial share of the global greenhouse gas emissions. The transition to net-zero emissions by 2050 demands development of industrial infrastructure and implementation of low-carbon technologies. This transition is expected to result in altered exposure scenarios for workers. This scoping review aims to map the evidence on the occupational exposures and related health consequences of industrial decarbonization processes and to identify current research gaps. The review will follow the JBI and PRISMA-ScR guidelines for scoping reviews and is registered under the Open Science Framework (osf.io/7xzy4). A systematic literature search combining terms related to industrial decarbonization with occupational and worker-related terms will be conducted in Web of Science, Embase, MEDLINE, and Scopus. The search will be supplemented by targeted gray literature searches to identify relevant technical reports and policy documents. Data will be charted using a standardized template and synthesized descriptively and thematically by occupational exposure and health effects, investigated populations, industrial sectors, decarbonization processes or technologies to map patterns in the available evidence, identify emerging occupational risks, and current research gaps.

**Clinical trial registration:**

osf.io/7xzy4.

## 1 Introduction

Since the acceptance of the Paris Agreement on climate change in 2016, substantial progress toward climate neutrality has been achieved (1). Nevertheless, the transition is still too slow, placing demands especially on energy-intensive industries, e.g., cement, iron and steel, ferroalloy, chemical, and petrochemical industries, which currently account for 25% of the global CO_2_ emissions (1, 2). Fossil fuels are today used as energy sources, to achieve high temperatures and as feedstock in chemical processes, thus resulting in a high carbon footprint in these sectors. In addition, chemical transformation processes may themselves directly result in CO_2_ emissions, as the conversion of carbon-containing raw materials during industrial reactions can release greenhouse gases as process by-products (3). To achieve carbon neutrality by 2050, energy-intensive industries need modernized infrastructures, improved industrial processes, and implementation of low-carbon technologies (4). However, decarbonization of the production of cement, steel and chemicals is challenging or even technically impossible. Thus, carbon neutrality should be achieved primarily through sustained emission reductions via the decarbonization of energy systems, including the replacement of fossil fuels with renewable energy (green direct electrification, green hydrogen and biofuels) alongside improvements in energy efficiency. Carbon dioxide removal is additionally required to remove residual emissions from sectors that are difficult to decarbonize (5). Carbon capture, use and storage (CCUS), is thus important, as CO_2_ can be captured, stored or even transformed into other raw materials that can be used in chemical or cement industries (6–8). In the metallurgical industry, net carbon-reducing reductants, such as biocarbon, can also be used as alternatives to coke and coal (9). In most energy-intensive industries, a combination of strategies is required to reach the carbon neutrality goals. In addition, process improvements are critical to ensure optimal energy efficiency and preserved product quality.

As these industries are the backbone for modern economies, developing solutions that ensure a successful transition to carbon neutral, energy-intensive production is both necessary and actively supported by EU initiatives (10). While several studies have reviewed reductions in emissions, benefits on ecological systems and climate impacts (11, 12), occupational health implications following industrial decarbonization processes are poorly evaluated (13). The increased industrial use of biomass, biogas, and hydrogen for energy production may introduce new occupational health risks during production, transportation, and storage. In addition, the utilization of novel biocarbon sources, as well as the introduction of emerging technologies and processes may lead to new occupational exposures and exposure scenarios. Thus, although there is a concern that these industrial transitions may alter occupational exposure profiles, the consequences on worker health remain unclear. Systematic mapping of emerging chemical and biological hazards and consequences for worker health is therefore urgently needed.

To provide a comprehensive overview of occupational implications of industrial decarbonization, this scoping review aims to identify and map the available evidence on emerging chemical and biological exposures and associated worker health outcomes in energy-intensive industries undergoing decarbonization. The review will focus on decarbonization strategies involving (i) green hydrogen or biofuels as renewable energy sources, (ii) CCUS strategies for CO_2_ reduction, and (iii) net carbon-reducing reductants. Specifically, the review will map occupational exposures and health outcomes investigated, worker populations and industrial sectors represented, and decarbonization process or technology evaluated across the available evidence. Lastly, the review will identify key research gaps to inform future occupational health research. Electrification, automation, and artificial intelligence are outside the scope of this review, as its primary focus is on emerging chemical and biological occupational hazards and associated worker health outcomes.

## 2 Methods and analysis

This scoping review will be conducted in accordance with Joanna Briggs Institute (JBI) methodological guidance (14) and reported following the PRISMA-ScR guidelines (15). The protocol is registered in the Open Science Framework (osf.io/7xzy4). Drafting of protocol and search strategies was initiated in April 2026, and the scoping review is expected to be finalized by December the same year.

A scoping review design was selected due to the emerging and heterogeneous evidence on occupational exposures and health outcomes associated with decarbonization of heavy industries. Literature spans multiple sectors, technologies, and study designs, including emerging risks, which are conceptually diverse and not yet sufficiently mature for systematic review synthesis.

The following primary research question was defined: “What occupational exposures and potential health outcomes have been reported in decarbonization of energy-intensive industries?” The review is further guided by the following secondary questions: (i) “What occupational chemical and biological exposures and associated worker health outcomes are reported or assessed?”, (ii) “Which worker populations have been investigated?”, (iii) “What is the global distribution of energy-intensive industrial sectors and decarbonization processes represented in the available evidence?”, and (iv) “What key research gaps exist in the current evidence?”.

### 2.1 Eligibility criteria

Eligibility will be defined using the Population–Concept–Context (PCC) framework:

- Population: Workers and employees.
- Concept: Occupational chemical and biological exposures and related health outcomes arising from industrial decarbonization strategies including green hydrogen or biofuel implementation, CCUS strategies, and the use of net carbon-reducing reductants. The review focuses on occupational hazards introduced, modified, or mitigated by these strategies and does not include studies addressing only public health concerns, environmental emissions, climate impacts, engineering performance, or process efficiency without an occupational health relevance.
- Context: Energy-intensive industries globally, including sectors such as metallurgy, cement production, chemical manufacturing, oil and gas processing, and industrial energy production (e.g., industrial scale biomass-based power generation).

Documents and studies will be included if they (i) concern workers in energy-intensive industries, (ii) assess occupational chemical and biological exposures, report worker health outcomes, or mechanistic toxicological evidence related to industrial decarbonization strategies, and (iii) are conducted in metallurgy, cement, chemical manufacturing, oil and gas processing, or industrial energy production settings. Additionally, only sources published in English and available in full text will be included. Sources will be excluded if they (i) do not involve occupational settings or worker populations, (ii) focus exclusively on public health, environmental emissions, climate impacts, engineering performance, process optimization, or economical aspects without addressing occupational exposures or worker health, (iii) focus on sectors outside energy-intensive industry, (iv) use solely electrification, automation, or AI in decarbonization processes, (v) are editorials, commentaries, conference abstract without sufficient data, and (vi) are not available in English.

### 2.2 Search strategy and study selection

The search strategy was developed by a research librarian (IJ) and will be used in Web of Science, Embase, Medline, and Scopus databases. Publications will be uploaded to Covidence (https://www.covidence.org/) and automatically checked for duplicates. An example of a search strategy can be found in Table 1. Moreover, the reference lists of full-text articles will be scrutinized to identify any further eligible studies (backward citation search). Title–abstracts and full texts will be screened independently by two reviewers. Disagreements will be resolved through discussion or through consultation with a third reviewer. The screening and selection process will be shown in a PRISMA-ScR flowchart.

**Table 1:** Example of search strategy (Web of Science).

No (#)	Search strategy: Web of Science
1	TS = (decarboni^*^ OR “carbon capture” OR “carbon reduction” OR “CO_2_ reduction” OR “greenhouse gas reduction” OR “emission reduction” OR “net zero” OR “low-carbon” OR “low-emission” OR “fossil-free” OR “green transition” OR Biofuel OR Biomass OR Biocarbon OR Reductant^*^ OR charcoal OR biocoke OR hydrogen)
2	TS=(occupation^*^ or employee^*^ or worker^*^)
3	#2 AND #1

^*^ indicates search for multiple forms of the same root word

To capture emerging and non-indexed evidence, a complementary gray literature search will be performed to identify relevant policy, technical, and institutional documents on occupational exposures and health outcomes associated with industrial decarbonization processes. Targeted searches will be performed based on the PCC framework on the official websites of relevant occupational health and safety organizations, international and European policy organizations, and energy and industrial transition organizations, Table 2. These sources were selected based on their relevance to occupational health, industrial decarbonization, energy transition strategies, and emerging workplace risks. For each search, the first 50 results will be screened for relevance in order of appearance by a single reviewer. Searches will be conducted over a predefined period, and the date of searches and identified records will be documented. The full text of the potentially relevant sources will be retrieved and evaluated in detail against the inclusion and exclusion criteria by two independent reviewers.

**Table 2:** Gray literature searches.

Category	Organization	Website
Occupational health and safety organizations	European Agency for Safety and Health at Work (EU-OSHA)	https://osha.europa.eu/en
	International Labor Organization (ILO)	https://www.ilo.org
	World Health Organization (WHO)	https://www.who.int
	National Institute for Occupational Safety and Health (NIOSH)	https://www.cdc.gov/niosh
	Institut national de recherche et de sécurité (INRS)	https://www.inrs.fr
	National Research Center for the Working Environment (NFA/NRCWE)	https://nfa.dk
	Finnish Institute of Occupational Health (FIOH)	https://www.ttl.fi/en
	Federal Institute for Occupational Safety and Health (BAuA)	https://www.baua.de
	Health and Safety Executive (HSE)	https://www.hse.gov.uk
	Netherlands Organization for Applied Scientific Research (TNO)	https://www.tno.nl/en/healthy/work-health/
	Central Institute for Labor Protection – National Research Institute (CIOP-PIB)	https://www.ciop.pl/en
International and European policy organizations	Organization for Economic Co-operation and Development (OECD)	https://www.oecd.org
	United Nations Economic Commission for Europe (UNECE)	https://unece.org
	European Union	https://european-union.europa.eu
Energy and industrial transition organizations	International Energy Agency (IEA)	https://www.iea.org
	International Renewable Energy Agency (IRENA)	https://www.irena.org
	Clean Hydrogen Partnership	https://www.clean-hydrogen.europa.eu
	World Steel Association	https://worldsteel.org
	Global Cement and Concrete Association (GCCA)	https://gccassociation.org

### 2.3 Data extraction and charting

Data from included studies and documents will be extracted by two reviewers using a data charting form developed by the reviewers, Table 3. Extracted information will include bibliographic information, study characteristics, industrial context, worker populations, occupational exposures or hazards, health outcomes, key findings, and identified research gaps. Prior to full data extraction, the data-charting form will be piloted on a selection of included studies representing different study types and industrial sectors by two reviewers independently. The data charting form will be refined to ensure that all relevant information is captured consistently. Any modifications to the data-charting form will be documented. Data presentation will be made descriptively and thematically. Data will be summarized according to occupational exposure type, and reported health outcomes, worker populations and industrial sectors, decarbonization processes and technologies. Results will be summarized narratively and presented in tabular form or graphically as frequency counts or percentages to map the existing evidence of emerging occupational risks and identify current knowledge gaps.

**Table 3:** Data extraction table.

Bibliographic information	Authors; year; country; publication type
Study characteristics	Study design; objectives
Industrial context	Industrial sector; process or activity; decarbonization technology or intervention; stage of implementation (if reported)
Population	Occupation(s); sample characteristics
Occupational exposures and hazards	Type of hazard; exposure assessment method; exposure measurements
Health outcomes	Outcome(s); assessment method; reported findings
Key findings	Main findings relevant to the review question(s)
Research gaps	Gaps and recommendations identified by the authors

## 3 Discussion and conclusions

Industrial decarbonization to meet carbon neutrality goals entails changes in industrial processes and the implementation of low-carbon technologies, leading to new occupational exposure scenarios and potential health concerns for workers. Given that evidence is dispersed across industrial sectors, technologies, and study designs, a scoping review is an appropriate methodology to systematically map the available evidence and highlight knowledge gaps.

The protocol outlines a transparent and robust methodology developed in accordance with the JBI guidelines for scoping reviews and will be reported following the PRISMA-ScR guidelines. The protocol is further strengthened by a comprehensive search strategy including multiple databases and a targeted gray literature search. Despite these strengths, limiting the review to English-language sources and the rapid changes in the field may result in relevant evidence being overlooked. Furthermore, while the scoping review methodology is suitable to address this broad and heterogenous topic, it does not include a formal assessment of risk of bias, nor a quantitative synthesis of the available evidence.

The completed scoping review will provide a comprehensive overview of the available literature, identify emerging occupational exposure scenarios and evidence gaps, and inform future occupational health research and occupational health and safety considerations during the transition toward low-carbon industries.
